# Parents perceptions of stress in a neonatal intensive care unit in Rwanda

**DOI:** 10.4102/curationis.v38i2.1499

**Published:** 2015-12-03

**Authors:** Priscille Musabirema, Petra Brysiewicz, Jennifer Chipps

**Affiliations:** 1 School of Nursing and Midwifery, College of Medicine and Health Sciences, University of Rwanda, Nyarugenge Campus, Rwanda; 2 School of Nursing and Public Health, University of KwaZulu-Natal, South Africa; 3 School of Nursing, University of the Western Cape, South Africa; 4 Sydney Nursing School, University of Sydney, Australia

## Abstract

**Background:**

Having a newborn infant hospitalised in the neonatal intensive care unit (NICU) is an unexpected and stressful event for a family. A number of potential stressors to which family members of patients in these units may be exposed have been identified, although no studies about this issue have been conducted in Rwanda.

**Aim:**

The aim of this study was to describe and analyse parental perception of stress that resulted from having their infant admitted to a NICU in Kigali, Rwanda.

**Method:**

A quantitative survey was used to describe and analyse parents’ perceptions of stress when they had an infant admitted to a NICU. The Parental Stress Scale: Neonatal Intensive Care Unit was used to measure the level of stress that those parents experienced.

**Results:**

The results indicated that parents experienced stress from having their infants cared for in a NICU. The most stressful events were the appearance and behaviour of the baby with a mean score of 4.02, whilst the subscale items related to sights and sounds were found to be the least significant source of stress for parents with a mean score of 2.51. In addition, the current study found that parents’ age, educational level, occupation, and infant birth weight were associated with parental stress.

**Conclusion:**

The study established that a range of factors was responsible for parental stress when a baby was cared for in a NICU. Identification of these factors could enable health professionals from a hospital in Kigali, Rwanda, to facilitate parents’ adjusting and coping.

## Background

A number of potential stressors to which family members of patients in a neonatal intensive care unit (NICU) may be exposed have been identified in the literature. The complexity of the physical environment of a NICU, the infant’s physical appearance and behaviour, staff and parent interaction and adjustments in the parents’ role have specifically been noted to be potentially stressful for parents with infants in a NICU (Trombini *et al.* 2008). In addition, the distress that these parents experience has been noted to influence their parenting behaviour (Lefkowitz, Baxt & Evans 2010) and can affect the long-term relationship with their children and their ability to take care of them (Carter *et al.* 2007).

In Rwanda there is only one hospital with a NICU where newborn babies with health problems are cared for. In the Rwandan culture, when a baby is born, everyone in the family is excited and ready to receive the newborn baby at home. The very first ‘official’ social function in the family to recognise the homecoming of the infant is the naming ceremony, commonly known as *Kurya ubunnyano*, which is held on the eighth day after the birth of a child (Nsanzimana 2012). In the event that the baby is admitted to a NICU, the routine ceremonies are not held and all family members may become demoralised and worried about the health of the infant.

A variety of factors, including personality, mental health, family, as well as social and pregnancy-related events are likely to contribute to the type and magnitude of stress that parents of an ill neonate experience. These factors have been noted to contribute to parental stress such as family functioning, socio-economic status, parent perceptions of infant illness, high trait anxiety, and available sources of support (Busse *et al.* 2013; Holditch-Davis & Miles 2000; Singer *et al.* 2010). Furthermore, the length of stay of the infant in a NICU is thought to have an effect on parental stress and it has been demonstrated that the longer the baby is hospitalised, the higher the level of stress, specifically for fathers, with regard to their communication with the staff (Harvey 2010). In Rwanda, the researcher’s own experience indicates that parents with infants in a NICU are challenged by the policies and regulations in respect of family access to their babies and Hurst (2004) emphasises the need for parents to have access to their babies. Restricting visiting hours reduces the ability to assume the parental role and creates a separation between the infant and parents (Bruns & Klein 2005). It is recognised that poor staff-family interaction does not only influence the baby’s care negatively, but may also be a source of distress and discontent for the parents (Orzalesi & Aite 2011).

## Problem statement

Having a newborn infant hospitalised in a NICU is an unexpected and stressful event for the family (Busse *et al.* 2013). The NICU is a specialised area that provides medical treatment and nursing care either for infants born prematurely, or for seriously ill newborn infants. Under normal circumstances the process of parent-infant bonding occurs within the first few days after birth and creates the basis for a lifelong relationship between the parents and their offspring (Gooding *et al.* 2011). Parent-infant bonding can be affected when the baby is born prematurely or experiences health risk, which necessitates admission to a NICU. The admission of a newborn baby to a NICU in conjunction with other stressors of being in ICU could cause substantial stress for the parents. Reducing parental stress and anxiety during the infants’ hospitalisation in a NICU should be a key nursing support function to enhance parental skills in challenging situations.

Although cross-cultural studies that investigate parental responses to their child’s hospitalisation have been conducted (Krulik *et al.* 1999; Lau *et al.* 2007), parental perception of stress in a NICU in the Rwandan context lacks investigation.

The aim of this study was to describe and analyse parental perception of stress that resulted from having their infants admitted to a NICU in Kigali, Rwanda. The Parental Neonatal Intensive Care Unit Stress Model (Wereszczak, Miles & Holditch-Davis 1997) was the conceptual framework chosen to guide this study.

## Research objectives of the study

The objectives of the study were:

- To identify situations Rwandan parents describe as stressful whilst in a NICU environment.
- To describe the challenges faced by Rwandan parents when an infant is admitted to a NICU.
- To analyse parents’ and infants’ characteristics that might contribute to the parental stress in a NICU.

## Definition of key concepts

*Stress:* Stress is defined as ‘psychological and physical strain or tension generated by physical, emotional, social, economic, or occupational circumstances, events, or experiences that are difficult to manage or endure’ (Colman 2003:711). In this study, stress refers to psychological and physical strain or tension experienced by a parent whose infant has been admitted to a NICU as measured by the Parental stress scale: Neonatal intensive care unit (PSS: NICU).

*Stressor:* A stressor is defined as physical, psychological or social force that puts real or perceived demands on the body, emotions, mind, or spirit of an individual (Colman 2003). In this study, a representative list of potential stressors refers to NICU sights and sounds, the way the baby appears and behaves, an adjustment in the parental role, and interaction with staff.

## Contribution to the field of study

Identifying the stressors parents experience could assist health professionals in a NICU with planning suitable interventions that are more family centred. In nursing practice, the findings of this study will assist with raising health professionals’ awareness about parents’ stressors in a NICU in Rwanda; it will also assist them with providing the relevant support to the parents who have a child in a NICU. At educational institutions, it will assist educators in emphasising possible gaps in the current nursing curriculum that need to be addressed.

## Literature review

Newborn babies born prematurely or the ones who experience medical complications are admitted to NICUs. Hall (2005) explains that being a parent of a newborn baby in a NICU is like being in another world; it is very different from what the parents have known and experienced before. Sights and sounds of the unit, infant appearance, parent-infant relationship and/or parental role and staff communication are emphasised as NICU environmental stressors (Wereszczak *et al.* 1997). The literature review describes the stressors that parents experience in this situation and the factors that contribute to the situation.

### NICU environment stressors

In a NICU, the baby is exposed to various forms of technology with disturbing sights and sounds. They are attached to many machines that measure heart rate and oxygen levels and these machines are beeping and buzzing around them. The environment in a NICU could, therefore, become very overwhelming to the baby, and also to the parents (Carter *et al.* 2007; Singer *et al.* 2010).

The physical appearance of the infant in a NICU and the perception that the baby is in pain may result in parental distress. The sights, sounds, and general environment of a NICU often cause stress and panic. Along with these issues, parents often experience difficulties with caregiving competencies and communication concerns related to the care of the infant. Furthermore, caregiving concerns frequently relate to parents’ apprehension upon discharge of their infants from a NICU.

The feeling of disruption of the parental role also contributes greatly to the amount of stress of parents in a NICU (De Rouck & Leys 2009). Recent studies using the PSS: NICU are consistent in their findings that indicate the change in parental roles is an important aspect behind parental stress in NICU settings (Carter *et al.* 2007; Dudek-Shriber 2004; Lau *et al.* 2007; Preyde & Ardal 2003; Shaw, Ikuta & Fleisher 2006). Interaction between parents and their infants is regarded as a crucial activity that has a significant impact on the parental care of a child, therefore, it directly relates to parental roles in NICU settings. Shaw *et al.* (2006) explore the influence of psychological distress amongst parents of infants in a NICU and report a strong correlation between severe presentation of acute stress disorder and parental role alteration. Another source of stress for mothers documented by Phillips and Tooley (2005) is the distress they often feel when they do not see, touch, or are not in close proximity of their babies. Understanding possible factors associated with parental stress is a fundamental principle for providing quality care that responds to the parents’ needs in such a way that it reduces stress and anxiety in a NICU.

A study conducted by Dudek-Shriber (2004) investigating factors related to altering parental roles experienced by parents in NICUs identifies that younger parents and parents of infants who suffer from a heart disease score the highest stress, related to change in parental roles. Several studies about parental stress that is the consequence of staff-parent communication in a NICU have been conducted (Browne & Talmi 2005; Nugent *et al* 2007). Browne and Talmi (2005) report that when parents are not provided with communication and information about the needs, care, and physical appearance of their infants, their stress levels increase. The same observation is revealed in the research study conducted by Harvey (2010) where ineffective staff communication is identified as a source of the highest level of parental stress.

Related to the parental stress factors in a NICU, language and cultural barriers are indicated to play a role in the amount of stress a family might experience whilst having an infant in the unit. Language barriers prevent families from accessing pertinent information about their infants (Denney *et al.* 2006). The study by Denney *et al.* (2006) regarding the levels of parent stress reveals that communication and linguistic barriers in a NICU environment yield high stress levels for parents. Underlining the importance of communication, the high demands placed on hospital professionals often result in a lack of time to communicate with the families (De Rouck & Leys 2009). Furthermore, the technological environment of the intensive care unit presents a challenge for the staff to facilitate a collaborative relationship with families whilst monitoring and caring for their critically ill infants (Johnson 2008).

### Factors that influence NICU stressors

Parental stress is in fact exacerbated by multiple factors such as personal characteristics of parents, situational factors, personal resources, and support in a NICU environment.

#### Personal characteristics

The personal characteristics of the parent, such as their past experiences and their personality, may influence parental stress. Literature in support of the link between parental stress and past experiences includes the developmental theory of continuity, whereby past experiences and an individual’s adjustment to these experiences are thought to influence later psychological functioning (Obeidat, Bond & Callister 2009). Positive past experiences are thought to promote later healthy functioning. Conversely, unpleasant past experiences are thought to increase an individual’s vulnerability to stress (Steedman 2007). Holditch-Davis and Miles (2000) similarly state that there are indications that past experience is a contributing factor to the level of stress experienced in a NICU.

When considering the quality or style of parenting displayed by an individual’s parents, two parental qualities that have been consistently identified in the literature are care and overprotection (Parker, Barret & Hickie 1992; Parker, Tupling & Brown 1979; Steedman 2007). Care is defined as ‘... affection, emotional warmth, empathy, and closeness’ (Parker *et al.* 1979:8) and overprotection control as ‘... intrusion, excessive contact, infantilisation [*sic*], and prevention of independent behaviour’ (Parker *et al.* 1979:8). Care appears to be the stronger construct of the two, as it accounts for more of the variance in a factor analysis undertaken by Parker *et al.* (1979). Either care or overprotection in an individual’s childhood is linked to the level of stress when such an individual becomes a parent (Assel *et al.* 2002; Carter *et al.* 2007). Assel *et al.* (2002) described the constructs of ‘warm responsiveness’ and ‘restrictiveness’ of parents, which correspond to care and over protection respectively. Structural equation modelling reveals significant pathways of influence. Mothers who have been exposed to the harsh and neglecting parenting qualities of their parents experience higher levels of stress and display lower levels of warmth towards their own children (Assel *et al.* 2002; Carter *et al.* 2007).

Other studies explain how personality may influence parental stress (Carter *et al.* 2007; Mulder *et al.* 2006). These studies conclude that personality dimensions are predictors of psychopathology, including neuroticism, anxiety, and depression.

#### Situational factors

Once an infant is admitted to a NICU, many factors account for parental stress. According to Denney *et al.* (2006), the birth of a newborn baby that results in the admission to a NICU is the initial stressor for a family. Parents fear for the survival of their infant and begin experiencing feelings of worry, depression, and anxiety (Obeidat *et al.* 2009). Similarly, the noticeable influence of the infant’s condition on parental stress is also reported (Carter *et al.* 2007; Dudek-Shriber 2004). The medical condition of an infant and the required medical/surgical treatment might directly increase parental stress (Carter *et al.* 2007).

Families may be faced with prolonged periods of stress, anxiety, and may often experience depression during the course of their child’s stay in a NICU. Indeed, the environment may affect parenting abilities, with adverse influences on the family unit, bonding, and the infant’s long-term developmental outcomes. Adding to these difficulties, at the time a premature baby is admitted to a NICU, there is often uncertainty about the baby’s prognosis. This is explained by the fact that the babies in a NICU are often in a critical condition. Parents are often very aware of the fact that these infants are at a higher risk of dying.

#### Personal resources

Wereszczak *et al.* (1997) explain personal resources of the parent as being family support, cognitive and financial resources. There is some indication in the literature that parents of ill neonates experience stress relating to matters such as family functioning, socio-economic status, and available sources of support (Carlson, Sampson & Sroufe 2003; Carter *et al.* 2007; Elgar *et al.* 2004; Martins & Gaffan 2000; Pinelli 2000). Research conducted by Carter *et al.* (2007) concludes that the high stress of parents with infants admitted to a NICU is associated with a lower household income and lack of family support.

## Research method and design

### Design

A quantitative survey was conducted to investigate parents’ perceptions of stress when their infants were admitted to a NICU, using the parental NICU stress model (Wereszczak *et al.* 1997) as the underlying framework.

### Context of the study

This setting of the study was a NICU at a tertiary hospital in Kigali, Rwanda, with a 200 bed capacity and 6 NICU beds. The average number of admissions to that NICU during the nine months prior to this study was 13 patients per month with a length of stay of approximately two weeks per infant.

### Population and sampling

The population was 110 parents of infants admitted to a NICU at a tertiary hospital in Rwanda. Inclusion criteria for this study required parents to have an infant admitted to a NICU, to agree to participate, and the condition of the child had to be determined by the medical staff as stable to exclude any confound cause of additional stress for the parents. No sampling was carried out and all parents of children admitted to a NICU at a tertiary hospital in Rwanda at the time of data collection over a period of eight months (from August 2011 to March 2012) were included.

### Materials

Data were collected using an existing 46 item self-report questionnaire, namely the PSS:NICU developed by Miles and Funk in 1987. This questionnaire has a well-established reliability and validity (Miles, Funk & Carlson 1993; Reid & Bramwell 2003). The PSS:NICU is a widely used tool to evaluate parental perception of stress experienced during NICU hospitalisation, that is, the experience which has caused parents to feel anxious, upset, or tense (Miles & Funk 1987). This scale identifies parental stress sources: the NICU physical environment, the change in parental roles, and the infants’ appearance. Each item in the PSS:NICU asks the parent whether or not he or she has experienced a particular situation, for example, seeing the baby with tubes and equipment on or near him or her. Those parents who have had such an experience are asked to rate its stressfulness on a scale from 1 (not at all stressful) to 5 (extremely stressful). In addition to the Miles and Funk (1987) tool, the researcher added items that were related to the demographic characteristics of parents and infants in Rwanda. Permission to translate and use the tool in the Kinyarwanda language from English was obtained from the original developer. A language expert at the Kigali Health Institute translated the English PSS:NICU into Kinyarwanda and then back translated into English to ensure the meaning was not altered.

## Validity and reliability

The psychometric properties of the PSS:NICU were originally evaluated in the USA and Canada by Miles *et al.* (1993) and was further assessed in studies by Franck *et al.* (2005), and Reid and Bramwell (2003). Internal consistency was good with alpha coefficients of 0.70 for each subscale in the original PSS:NICU and internal consistency for the total instrument ranged from 0.94 to 0.89. Inter-scale correlations showed moderate to strong correlations between the subscales and between the subscales and the total scores. The construct validity of the PSS:NICU was measured and found significant by comparing the scale scores with the scores of the state anxiety scale of State-Trait Anxiety Inventory (STAI) (Spielberger *et al.* 1983).

To ensure the validity and reliability of the PSS:NICU for use with Rwandan parents, a pilot study with four parents of infants admitted to a NICU had been conducted. The pilot study was carried out one week before the final study. The gaps revealed by results of that pilot study included misunderstood terms that respondents interpreted differently and unclear statements that would not generate required information accurately. These editing issues were addressed by the researcher by ensuring that the meaning of the language used was clear.

## Data collection method

After obtaining permission to conduct the research from the Kigali Health Institute and the hospital administration, an appointment was made with the NICU manager. The parents were approached when they came to visit their children. Eligible parents were informed about the purpose of the study and asked to sign informed consent forms before they were allowed to participate in the study. All those parents who gave consent were asked to complete the questionnaires when it was convenient for them. The researcher collected the completed questionnaires on the day of the parents’ visit to the NICU. Data were entered into a secure Statistical Package for the Social Sciences (SPSS) database.

## Data analysis

All data were analysed using the SPSS version 16.0 and 21 computer program. Missing or incomplete data were excluded from the analysis. The data were arranged into subgroups of items that allowed a more meaningful examination of different dimensions. Descriptive statistics were used and results were expressed in the format of absolute numbers and percentages for nominal level data, and in mean values and standard deviations for continuous variables.

The PSS:NICU instrument can be scored either by calculating the frequencies of stressful experiences or by calculating two total scores. Metric 1: *Stress occurrence level* measures the level of stress produced when a situation occurs (in this study, only cases of parents who reported a stressful situation). Metric 2: *Overall stress level* measures the general stress from the environment (in this study, all cases whether exposed to a stressful situation or not) (Miles & Funk 1987). Metric 2 is used to describe the levels of stress parents have experienced whilst having an infant in a NICU. When the focus is on the NICU environment, Metric 1 (*Stress occurrence level*) is more useful to obtain a better understanding of the amount of stress each aspect of the environment causes (Miles & Funk 1987).

Scale scores were calculated by averaging those stress responses for the items on each scale and for the total scale. Independent samples obtained through the Kruskall Wallis and Mann Whitney U tests were used to examine the association between the characteristics of the parents and infants and the three general stress constructs of the PSS:NICU instrument, that is, infant appearance and behaviour, sights and sounds in the NICU, and adjustment in parental role.

## Results

There were 110 parents approached for the study with 98 responding. Of the parents who responded, 24 were fathers and 74 were mothers. Twelve potential respondents chose not to participate, citing time constraints as the reason for declining the invitation to participate.

### Description of sample

Respondent ages ranged from 21 to 55 years, with the majority 38.8 % (*n* = 38) in the age range of 31 to 35, followed by 22.4% (*n* = 22) in the age range of 26 to 30 years. The mean respondent age was 31.98 years. Nearly all the respondents were married (96), were employed, with the majority (62.2%, *n* = 61) being publically and privately employed, and all respondents were educated with a marginal majority (52%, *n* = 51) at university level.

Most of the infants (90; 91.9%) were premature (< 36 weeks) with three extremely premature (< 28 weeks); the remaining eight were full term babies (> 36 weeks). The mean gestational age of the infants was 31.78 weeks (SD 3.1). As a result of the prematurity, most infants had an extremely low birth weight ranging from 600 g to 3.6 g, with a mean birth weight of 1.285.5 g (SD 737.8).

### Stressful situations in a NICU

Based on the literature review and the NICU stress framework, situations that parents found stressful were reported by using two constructs: the appearance and behaviour of the baby, as well as the sights and sounds in the NICU.

#### Baby’s appearance and behaviour

Respondents rated this construct the highest with an average stress score of 3.8 SD 0.39 (95% CI 3.7–3.8) and an actual stress occurrence average score of 4.2 SD 0.31 (95% CI 4.2–4.3) out of a possible 5. The most stressful perceived behaviour occurred when a baby stopped breathing; all parents reported extreme stress responses. That was followed by a baby with a limp and weak appearance (93; 94.9%); 72 of those parents reported extreme stress reactions (Table 1).

**TABLE 1:** Parents’ perception of how their babies appeared and behaved (*n* = 98).

Perceptions	Stressed by event *n* (%)	General stress score (95% confidence intervals)
Stop breathing	98 (100)	5.0
Limp and weak	93 (94.9)	4.5 (4.2–4.7)
Jerky or restless movements	94 (94.9)	4.4 (4.2–4.6)
Abnormal breathing patterns	98 (100)	4.3 (4.1–4.5)
Bruises, cuts, or incisions	96 (98)	4.2 (4.0–4.3)
Pain	89 (90.8)	4.1 (3.8–4.4)
Intravenous feed line or tube	92 (93.9)	4.0 (3.8–4.2)
Do not cry like other babies	82 (83.7)	4.0 (3.6–4.3)
Baby looked sad	90 (90.8)	3.9 (3.6–4.2)
Small size	85 (86.7)	3.8 (3.5–4.2)
Crying for long periods of time	89 (90.8)	3.8 (3.5–4.1)
Wrinkled appearance	83 (84.7)	3.8 (3.5–4.1)
Tubes and equipment	88 (89.8)	3.8 (3.5–4.0)
Needles and tubes inserted	82 (83.7)	3.6 (3.3–3.9)
Suddenly change colour	72 (73.5)	3.3 (2.9–3.7)
Baby’s unusual colour	72 (73.5)	3.2 ( 2.8–3.6)
Looks afraid	5 (5.1)	0.3 (0.03–0.5)

#### Sights and sounds in the NICU

Respondents rated this construct the second highest with an average stress score and actual stress occurrence score of 2.7 SD 0.7 (95% CI 2.6–2.9) out of a possible 5. Generally, the most stressful events were sudden noises or alarms (93; 94.9%) (Table 2), with 41 respondents who reported being extremely stressed. A significant number of parents (79; 80.6%) experienced stress when they saw a respirator or ventilator being used on a baby (Table 2).

**TABLE 2:** Parents’ perceptions of sights and sounds in the NICU (*n* = 98).

Perceptions	Stressed by event *n* (%)	General stress score (95% confidence intervals)
Sudden monitor alarms	93 (94.9)	4.0 (3.7–4.2)
Having respirator for baby	79 (80.6)	3.6 (3.3–4.0)
Large number of staff	97 (99)	2.4 (2.0–2.7)
Noises of monitors and equipment	41 (41.8)	2.2 (1.9–2.5)
Others sick babies	46 (46.9)	2.2 (1.9–2.5)
Monitors and equipment	39 (39.8)	2.1 (1.8–2.4)

### Challenges faced by parents

Generally, parental role adjustments were rated more stressful than staff communication with an average stress score and actual stress occurrences average score of 2.3 SD 0.5 (95% CI 2.2–2.4) out of a possible 5 compared to 2.1 (2.0–2.1). In the main, the most stressful challenge for parents 98% (*n* = 96) was being separated from their babies. Not being able to share the baby with other family members was shown not to be stressful by 93.9% (*n* = 92) of the respondents (Table 3).

**TABLE 3:** Parents’ perceptions of parental role adjustment stress (*n* = 98).

Perceptions	Stressed by event *n* (%)	General stress score (95% confidence intervals)
Separation	98 (100)	5.0 (4.9–5.0)
Cannot provide care	71 (72.4)	3.0 (2.7–3.3)
Cannot hold baby when I want	68 (69.4)	2.8 (2.5–3.2)
Afraid of touching or holding	67 (68.4)	2.8 (2.4–3.1)
Feel helpless and cannot protect	69 (70.4)	2.6 (2.4–2.9)
Feeling helpless in terms of assistance	68 (69.4)	2.6 (2.3–2.9)
Cannot feed	44 (44.9)	2.0 (1.6–2.4)
No time to be alone with baby	58 (59.2)	1.8 (1.7 0 2.0)
Cannot share baby with family	6 (6.1)	1.2 (1.1–1.4)
Feeling staff is closer to my baby than I am	12 (12.2)	1.2 (1.1–1.4)
Forgetting what baby looks like	-	-

In terms of staff communication, the most stressful issue was staff not sharing enough information about tests and procedures (96; 98%), not conversing with parents frequently enough (89; 90.8%), and using too many words (80; 81.6%) (Table 4).

**TABLE 4:** Parents’ perceptions of staff factors causing stress (*n* = 98).

Perceptions	Stressed by event *n* (%)	General stress score (95% confidence intervals)
Not enough information about tests	96 (98.0)	4.6 (4.5–4.8)
Not conversing with parents frequently enough	89 (90.8)	4.1 (3.8–4.4)
Do not understand words	80 (81.6)	4.0 (3.6–4.3)
Not sure will be called about changes	19 (19.4)	1.3 (0.9–1.7)
Explain things too fast	11 (11.2)	0.5 (0.3–0.7)
Too many staff talking to me	-	-
Conflicting information about baby	-	-

### Association between parental and infant characteristics and general stress constructs

A significant association was found between parental age and general stress when confronted with an infant’s appearance in a NICU with the older age groups reporting higher stress levels (*K* = 13.6, *p* = 0.004). Parents with only primary school level education also reported significantly higher stress levels (3.2 SD 0.5) in terms of the sights and sounds of the NICU than university graduates (2.6 SD 0.7) (*K* = 8.8, *p* = 0.012). There was no significant association between the parents’ gender or marital status and the three constructs.

In terms of infant characteristics, perceived stress with regard to the parental role was higher for babies older than 36 weeks (*K* = 6.1, *p* = 0.046) and babies > 2500 g (*K* = 10.7, *p* = 0.013). Conversely, perceived stressed in relation to the appearance and the behaviour of a baby was higher in low weight babies < 1000 g (*K* = 9.8, *p* = 0.020).

## Ethical considerations

Ethical approval was obtained from the Kigali Health Institute administration after approval by the appropriate institutional review board. Permission had also been obtained from the hospital where the study was conducted. To protect those vulnerable respondents, the researcher carefully observed each family member she approached to ensure each one of them was not stressed by completing the questionnaire; as a precautionary measure, psychological support was available. The parents were approached when they came to visit their children and eligible parents were informed about the purpose of the study and written consent was obtained prior to participation in the study. Parents were assured that their participation was voluntary, data could not be traced back to them, they could withdraw from the study at any time, and their refusal to participate would in no way affect the care that their child was receiving. All those parents who gave consent were asked to complete the questionnaires as and when it was convenient to them. The researcher collected the completed questionnaires on the day that the parents visited their children.

## Discussion of results

Three components of the results will be discussed.

### Situations that Rwandan parents describe as stressful in a NICU environment

The two situations that parents describe as stressful are the baby’s appearance and behaviour, and the sights and sounds of the ICU. A baby’s appearance is more stressful; this finding concurs with the findings of previous studies that have identified the physical appearance of an infant admitted to a NICU as the most significant source of stress for parents (Denney *et al.* 2006). On the other hand, Dudek-Shriber (2004) is convinced that parents experience a small degree of stress as a result of how their babies look and behave. The differences that occur in findings may be explained by the fact that each NICU has its unique context. There may also be culturally significant differences between other contexts and this population of Rwandan parents.

In terms of the experiences of stress in relation to the sights and sounds in a NICU, similar results are noted by Carter *et al.* (2007) and Singer *et al.* (2010). These studies state that sights, sounds, and the general environment of the NICU often cause stress and panic in the parents.

### Challenges that parents face

The main challenge faced by parents is the perceptions of the parental role adjustments. Dudek-Shriber (2004) and Steedman (2007) agree that the subscale in which parents reported their greatest stress is the parent-infant relationship. This finding is consistent with results of another study conducted by Carter *et al.* (2007) which found that adjustments in the parental role were the most stressful aspect of having an infant in a NICU.

Communication with staff is another challenge for parents. Parents believe that the importance of communication cannot be overemphasised, as stress is caused by poor or not sufficient explanations, and using too many words.

### Association between parental / infant characteristics and general stress constructs

The effect of the educational level on the amount of stress that parents with an infant in a NICU experience has been explained in literature. Dudek-Shriber (2004) argues that parents with a high school education and higher education have higher stress frequency levels than parents who have not completed high school. These findings are consistent with the findings of this study. Given the scarcity of research in this area, this issue needs to be addressed further in order to explore the impact of educational level on the stress that parents with infants admitted to a NICU experience, especially in the cultural context of Rwanda. The findings of this study are consistent with the findings of a study conducted by Carter *et al.* (2007) which indicates that parents with low birth weight infants experience higher levels of stress than the ones with full term infants admitted to a NICU.

## Practical implications and recommendations

The sources of stress for parents with an infant admitted to a NICU have been explained and it is important for health professionals who practise in a NICU to be aware of these sources of stress in an attempt to reduce them where possible.

In-service education for nurses and doctors who work in a NICU can be implemented and the educational curriculum of nurses and doctors should also be interrogated in respect of these issues. In future studies, it may be appropriate to validate the usefulness of these components for Rwandan parents and further research, using a qualitative approach that could provide more detailed information about parental stress.

## Limitations

Generalisation of the current study findings is limited because data were collected from parents at one hospital and in one geographical location. In addition, the sample of this study was not representative of the entire population because of the nature of convenience sampling. Although self-report questionnaires are a valuable method in psychological research, sole reliance on such questionnaires is a limitation, as the potential for subjective bias exists; for example social desirability refers to the tendency of individuals to present themselves in a positive light according to social norms and values (Colman 2003).

## Conclusion

The results of this study provide important information for health professionals who are working at the NICU in Rwanda. This information can be used to look at ways to reduce stress and mitigate adjustment and coping with regard to this stressful situation. This objective may be achieved by preparing and educating parents about their infants’ tests, treatment, and general health condition. They should also be made part of the decision making about their infants. Priority should be given to train NICU staff with the aim of preparing and supporting parents in terms of the sights and sounds in a NICU. Policies that reduce parental separation from their infants would address a significant cause of stress for parents. All these strategies will assist with preventing or reducing stress for parents when their infants are admitted to a NICU
